# MEASUREMENT OF GERIATRIC SURGERY OUTCOMES IN A NATIONAL REGISTRY

**DOI:** 10.1093/geroni/igz038.1486

**Published:** 2019-11-08

**Authors:** Emily Finlayson

**Affiliations:** University of California San Francisco, San Francisco, California, United States

## Abstract

The American College of Surgeons National Quality Improvement Program started a “Geriatric Pilot” in January 2014. This project has already collected 19 additional older adult specialty variables in more than 60,000 patients undergoing operations. Twenty-six medical centers participate from across North America. The variables collect information in the domains of cognition, function, mobility and decision-making. Variables are collected in both the pre- and post-operative settings. It is clear that the quality of surgical care cannot be limited to the immediate hospitalization. The pilot has recently expanded its use of longer-term outcomes and has begun collecting 30-day outcomes of functional status and living location.

